# Multilocular cystic nephroma in a 15-month-old infant: Successful surgical management and 10-year follow-up

**DOI:** 10.1016/j.ijscr.2025.112086

**Published:** 2025-10-21

**Authors:** Saida Hidouri, Ghada Habachi, Sabrine Ben Ammar, Mohamed Ali Chaouch, Abir Kalai, Faten Letaief

**Affiliations:** a University of Monastir, Faculty of Medicine of Monastir, Tunisia; b LR12SP13, Department of Pediatric Surgery, Zaghouan Regional Hospital, Tunisia; c University of Tunis El Manar, Faculty of Medicine, Tunis, Tunisia

**Keywords:** Multilocular cystic nephroma, Pediatric renal tumor, Cystic kidney mass, Nephrectomy, Case report, Infant, Histopathology

## Abstract

**Introduction:**

Multilocular cystic nephroma (MLCN) is a rare benign renal tumor of uncertain etiology that affects primarily infants and young children. Clinically and radiologically, it can closely resemble malignant renal tumors such as cystic partially differentiated nephroblastoma (CPDN) and cystic Wilms tumors, which makes a precise preoperative diagnosis particularly challenging.

**Case presentation:**

We report the case of a 15-month-old male infant who presented nonspecific symptoms, including diarrhea, reduced appetite, and progressive abdominal distension. Physical examination revealed a failure to thrive and a well-defined and firm abdominal mass. Imaging studies demonstrated a multilocular cystic lesion in the left kidney without evidence of calcification, lymphadenopathy, or metastasis. Due to diagnostic ambiguity and the suspicion of malignancy, an open radical nephroureterectomy was performed. Gross pathology revealed a multiloculated mass filled with clear fluid, and histological evaluation confirmed the diagnosis of MLCN.

**Clinical discussion:**

This case underscores the difficulty in distinguishing MLCN from malignant cystic renal tumors based solely on imaging. Although MLCN is benign, the potential for misdiagnosis requires surgical intervention. Complete excision remains both diagnostic and curative. Long-term outcomes are excellent, with a low risk of recurrence or malignant transformation. In our case, the child recovered well after the operation and showed no evidence of recurrence after ten years of follow-up, highlighting the favorable prognosis with appropriate treatment.

**Conclusion:**

MLCN should be considered in the differential diagnosis of pediatric cystic renal masses. Despite its benign behavior, surgical resection is often required due to diagnostic uncertainty. This case illustrates the importance of a multidisciplinary approach in the evaluation of renal tumors in children and reinforces the need for long-term follow-up.

## Highlights

• Multilocular cystic nephroma (MLCN) is a rare benign renal tumor in infants that often mimics malignant cystic renal lesions radiologically, making preoperative diagnosis difficult.
• Definitive diagnosis of MLCN relies on histopathological examination following surgical excision, which also serves as curative treatment.
• This case demonstrates the excellent long-term outcome of MLCN post-nephrectomy, with no recurrence observed over a 10-year follow-up period.

## 1 Introduction

Multilocular cystic nephroma (MLCN) is a rare benign renal tumor that primarily affects young children, peaking between 6 months and 4 years [1]. Clinically, it can present a diagnostic dilemma due to its radiologic features with other cystic renal lesions, including malignant entities such as partially differentiated cystic nephroblastoma (CPDN) and cystic Wilms tumor [2]. MLCN is often discovered incidentally or during evaluation for nonspecific abdominal symptoms. Accurate preoperative diagnosis remains challenging, as imaging modalities may not reliably distinguish MLCN from malignant counterparts [3]. Other pediatric cystic renal masses, including mesoblastic nephroma and cystic dysplasia, may also enter the differential diagnosis, further complicating the preoperative evaluation. Therefore, the definitive diagnosis is often established through histopathological examination after surgical resection. This case is distinguished by a 10-year recurrence-free follow-up, which is rarely reported in the literature and adds significant value to understanding the prognosis of MLCN. In this report, we present a case of MLCN in a 15-month-old infant, according to the SCARE guidelines [4] describing the clinical, radiological, and histological features, as well as the challenges in differentiating it from other pediatric renal cystic lesions.

## 2 Case presentation

**Fig. 1:**
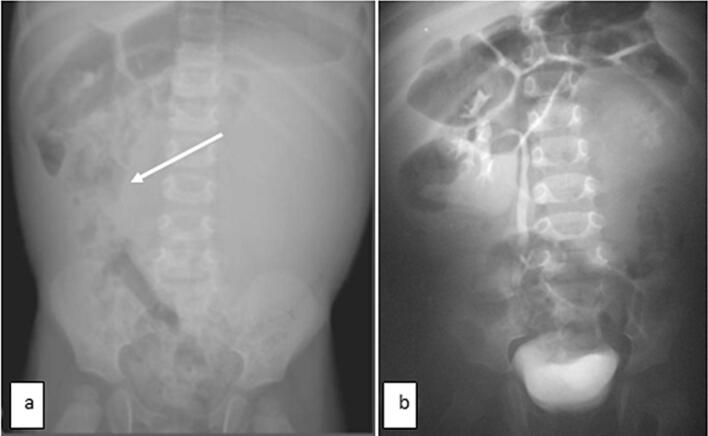
Radiological explorations. (a) Abdominal radiograph showing a left abdominal opacity that displacements the adjoining bowels (**white arrow**) (b) Intravenous pyelogram showing delayed left renal function with normal functioning right kidney.

**Fig. 2:**
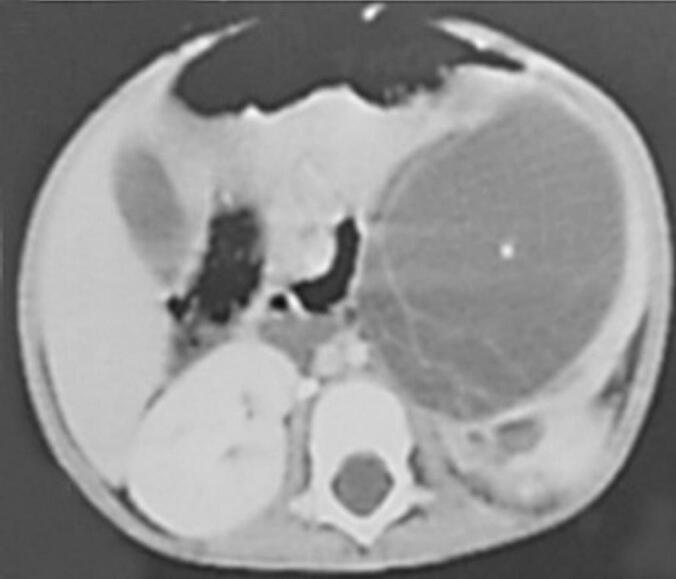
High-resolution abdominal CECT showing thin, contrast-enhancing septations without calcification or solid nodules.

**Fig. 3:**
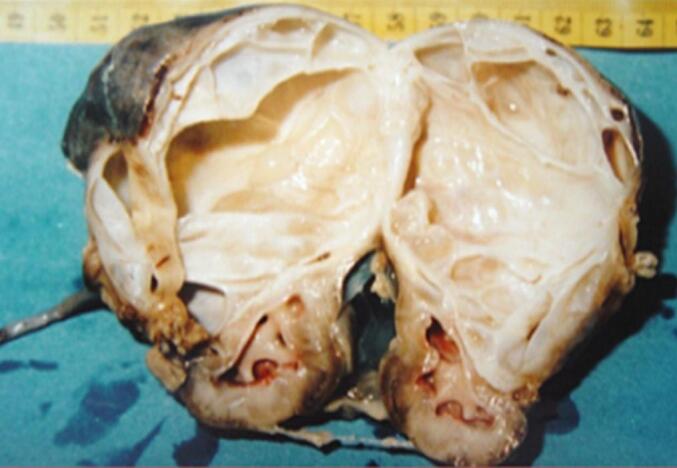
Gross examination showing a multilocular cystic tumor with fine septations.

**Fig. 4:**
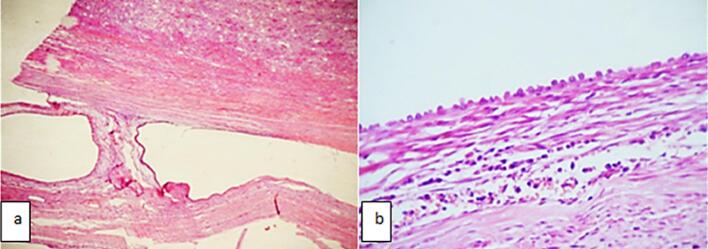
(a) Microscopic study showing optically empty cystic cells separated by fibrous septa (hematoxylin and eosin, original magnification x40) (b) Microscopic study showing a cubic tubular epithelium (hematoxylin and eosin, original magnification ×100).

As was seen in our healthy 15-month-old infant, referred to our department for a history of diarrhea, poor appetite, and abdominal mass. His antenatal and postnatal history was unremarkable. On examination, he showed failure to thrive, with a weight of 8.3 kg (−3 standard deviations), a blood pressure of 110/60 mmHg, and a 10 × 8 cm in the left hypochondrium and lumbar regions. It was firm, smooth, well-limited, and painless. Ultrasonography (US) revealed a large multilocular cystic mass in the left kidney, with no associated calcifications. An intravenous pyelogram indicated delayed secretion and no excretion from the left kidney, with a normal functioning right kidney. The intravenous pyelogram suggested delayed secretion and no excretion in the left kidney (Fig. 1). Abdominal contrast-enhanced computed tomography (CECT) showed a 7x8x7cm multilocular cystic mass in the left kidney with multiple internal septations and no lymphadenopathy or metastases (Fig. 2). Biology revealed isolated anemia (hemoglobin of 9.8 g/dl) with normal renal and liver function tests and normal urinary catecholamines. MLCN and CPDN were suspected, and an open radical nephroureterectomy was performed. The gross examination showed an intact capsule and a multiloculated lesion with clear fluid (Fig. 3), and histopathology confirmed the diagnosis of MLCN (Fig. 4). The child recovered uneventfully and was followed in an outpatient clinic annually. Ten years later, he remains asymptomatic with no recurrence.

## 3 Discussion

MLCN is a rare benign renal tumor characterized by multiple non-communicating cysts separated by a septa and surrounded by a fibrous capsule [1]. Although it can occur in both pediatric and adult populations, pediatric cases typically occur within the first two years of life and show a strong male predominance. The clinical presentation is often non-specific. Beyond CPDN and Wilms tumor, additional differential diagnoses include mesoblastic nephroma and cystic dysplasia, which can also present as multilocular renal lesions in infants. In our case, the patient exhibited symptoms such as diarrhea, poor appetite, and abdominal distention, with a palpable abdominal mass on physical examination. These symptoms are consistent with reported cases where abdominal mass is the most common presenting feature. Despite being benign, MLCN can reach large sizes, potentially leading to compressive symptoms or growth failure, as seen in our patient. Radiologically, distinguishing MLCN from other cystic renal masses, such as partially CPDN and cystic Wilms tumor, remains a challenge [2]. Both ultrasonography and contrast-enhanced computed tomography in our case revealed a multilocular cystic mass with septations, but neither provided definitive features to rule out malignancy. On imaging, MLCN typically shows thin, smooth septations without solid components or calcifications. CPDN often shows thicker septa with immature tissue, while a cystic Wilms tumor may present with irregular septations, solid nodules, and occasional calcifications. These overlapping features complicate preoperative differentiation. In our case, the septa were thin and smooth, without irregular thickening or nodularity, features that may favor a benign lesion such as MLCN. However, the absence of definitive radiological signs meant that the malignancy could not be excluded, highlighting the challenge of preoperative imaging in such cases. This highlights the limitations of imaging in preoperative diagnosis and reinforces the need for histological confirmation [3]. Histopathologically, MLCN is characterized with cysts lined by flattened or cuboidal epithelium and separated by fibrous septa, without immature elements. On the contrary, CPDN contains immature nephrogenic tissue, while Wilms tumor shows blastemal, stromal, and epithelial components [5]. The absence of these malignant features in our case confirmed the diagnosis of MLCN [5]. Histopathology of our patient confirmed the benign nature of the lesion, with no evidence of malignancy. Surgical excision remains the treatment of choice and is usually curative [6]. In our case, open radical nephroureterectomy was performed due to nonfunctionality of the affected kidney and suspicion of malignancy. Long-term follow-up is recommended, although recurrence is rare. Ten years after surgery, our patient remains asymptomatic with no signs of recurrence, underscoring the favorable prognosis associated with complete resection. Although nephron-sparing surgery is increasingly advocated in pediatric renal tumors, it was not appropriate in our case. The affected kidney did not show any function on the imaging, and the possibility of malignancy required complete excision to ensure oncological safety. Therefore, an open radical nephroureterectomy was performed. This case emphasizes the importance of considering MLCN in the differential diagnosis of cystic renal masses in infants. Although the lesion is benign, its clinical and radiologic similarities to malignant entities require a cautious approach, involving surgical excision and histopathological evaluation for a definitive diagnosis. Very few cases of MLCN with long-term follow-up beyond 5 years have been published, and recurrence has been extremely rare. Our 10-year follow-up further confirms the excellent prognosis associated with complete excision.

## 4 Conclusions

MLCN is a rare but important differential diagnosis in infants with cystic renal masses. Despite its benign nature, its clinical and radiological overlap with malignant tumors requires surgical resection and histopathological evaluation for definitive diagnosis. This case highlights the diagnostic challenges of MLCN, while also demonstrating an excellent long-term prognosis, as evidenced by our patient's 10-year recurrence-free follow-up.

## Consent

Written informed consent was obtained from the patient's parents/legal guardians for publication and any accompanying images. A copy of the written consent is available for review by the Editor-in-Chief of this journal upon request.

## Ethical approval

The study protocol was reviewed and approved by the Clinical Research Ethics Committee of the Faculty of Medicine of Monastir, University of Monastir (Ethical Committee reference number: NAC - 206). All procedures were carried out according to the Declaration of Helsinki and relevant national regulations. Written informed consent was obtained from the patient's legal guardians prior to participation, and all data were anonymized to preserve confidentiality.

## Funding

This research did not receive specific grants from the public, commercial, or nonprofit sectors.

## Author contribution

Saida Hidouri contributed to the conception and design of the case report, collected clinical data, and drafted the initial manuscript. Ghada Habachi participated in data interpretation, literature review, and critically revised the manuscript. Sabrine Ben Ammar was involved in patient management and provided key clinical details. Mohamed Ali Chaouch contributed to the critical revision of the manuscript for important intellectual content. Abir Kalai assisted with data analysis and imaging interpretation. Faten Letaief supervised the work, ensured the accuracy of the clinical content, and contributed to manuscript editing. All authors read and approved the final version of the manuscript.

## Guarantor

Mohamed Ali Chaouch, MD.

## Research registration number

N/A.

## Conflict of interest statement

No conflict of interest to disclose.
